# Folate-Based Radiotracers for PET Imaging—Update and Perspectives

**DOI:** 10.3390/molecules18055005

**Published:** 2013-04-29

**Authors:** Cristina Müller

**Affiliations:** Center for Radiopharmaceutical Sciences ETH-PSI-USZ, Paul Scherrer Institute, Villigen-PSI 5232, Switzerland; E-Mail: cristina.mueller@psi.ch; Tel.: +41-56-310-4454; Fax: +41-56-310-2849

**Keywords:** folate receptor, folic acid, PET, ^68^Ga, ^18^F, ^152^Tb, ^44^Sc, cancer, imaging

## Abstract

The folate receptor (FR) is expressed in many tumor types, among those ovarian and lung cancer. Due to the high FR affinity of folic acid, it has been used for targeting of FR-positive tumors, allowing specific delivery of attached probes to the malignant tissue. Therefore, nuclear imaging of FR-positive cancer is of clinical interest for selecting patients who could benefit from innovative therapy concepts based on FR-targeting. Positron emission computed tomography (PET) has become an established technique in clinical routine because it provides an increased spatial resolution and higher sensitivity compared to single photon emission computed tomography (SPECT). Therefore, it is of critical importance to develop folate radiotracers suitable for PET imaging. This review article updates on the design, preparation and pre-clinical investigation of folate derivatives for radiolabeling with radioisotopes for PET. Among those the most relevant radionuclides so far are fluorine-18 (t_1/2_: 110 min, E_av_β^+^: 250 keV) and gallium-68 (t_1/2_: 68 min, E_av_ β^+^: 830 keV). Recent results obtained with new PET isotopes such as terbium-152 (t_1/2_: 17.5 h, Eβ^+^: 470 keV) or scandium-44 (t_1/2_: 3.97 h, E_av_ β^+^: 632 keV) are also presented and discussed. Current endeavors for clinical implementation of PET agents open new perspectives for identification of FR-positive malignancies in patients.

## 1. Introduction

PET imaging has become a widely used technology in oncology in which it plays a crucial role in the diagnosis of cancer, staging of the disease as well as planning and monitoring of the therapy [1]. This is accomplished primarily through use of 2-deoxy-2-[^18^F]fluoro-D-glucose ([^18^F]FDG) among other ^18^F- and ^11^C-based metabolic radiotracers [1,2]. More recently PET radioconjugates of appropriate targeting agents which are specific for tumor-associated receptors have been introduced in clinical routine use. They may extend PET applications for *in vivo* quantification of receptor expression and its possible changes during the course of cancer therapy [2]. In this respect the most prominent examples are ^68^Ga-labeled somatostatin analogs (e.g., ^68^Ga-DOTATATE, ^68^Ga-DOTATOC) which have raised significant attention for PET imaging of neuroendocrine tumors [3,4,5].

Herein we present the folate receptor (FR) as a promising cell membrane-associated target for PET imaging of cancer (FR-α) and potentially also inflammatory diseases (FR-β) through use of folic acid-based radioconjugates [6].

### 1.1. Folate Receptor Targeting Strategy

Due to the existence of four distinct FR genes there are four FR proteins termed FR-α, -β, -γ and -δ. These FR-isoforms are polypeptides of 220–237 amino acids that share 68–79% sequence identity and contain eight conserved putative disulfide bonds [7,8,9,10,11]. The FR-α and the FR-β are glycosylphosphatidyl inositol-anchored membrane proteins which bind folates and folate conjugates with high affinity and internalize via endocytosis [7]. Importantly, the FR-α is overexpressed on a variety of tumor types but shows limited expression in normal tissue [12,13]. Among FR-α expressing malignancies are cancers of the ovaries, uterus, brain, lungs, kidneys, breast and colon-rectum where the frequency of positive cases lies between 32% and 90% [13,14]. The FR-β is overexpressed on activated but not resting macrophages that are involved in inflammatory processes such as for instance rheumatoid arthritis [15].

In normal healthy tissue the existence of the FR is limited to a few sites where it is expressed on the apical side of polarized epithelial cells in the lung, choroid plexus, salivary glands and the placenta [13,16,17,18]. Most important with regard to targeting applications are the kidneys where the FR is expressed in the proximal tubules [19,20,21].

The vitamin folic acid, emerged as an almost ideal targeting agent for imaging purposes and therapy of cancer and inflammatory diseases because of the very high affinity (K_D_ < 10^−9^ M) to both, the FR-α and the FR-β and because of its non-toxic and non-immunogenic properties [22]. With regard to the development of radiotracers, further advantages of using folic acid are its accessibility for chemical modification and its robustness against elevated temperatures which are often required for radiolabeling procedures.

### 1.2. Folate-Based Radiotracers

Folate-based radiotracers may be of interest since FR-α expression levels are reported to correlate with the aggressiveness of particular cancer types. In non-small cell lung cancer patients higher levels of FR-α expression was reported to correlate with an increased survival [23]. In contrast, a significant correlation or at least a trend for correlation was determined between the FR-expression level (detected by immunohistochemical analysis) and a reduced survival time in ovarian, endometrial, breast and primary colorectal cancers [24,25,26]. More recently, the FR was also identified as a marker for prediction of the survival rate in hepatic colorectal cancer metastases [27].

The level of FR-β expression on macrophages involved in inflammatory processes was found to correlate with the production of reactive oxygen species and hence the FR-β was determined as an activation marker for macrophages [15]. Due to a direct correlation between the level of macrophage activity and the observed joint inflammation in rheumatoid arthritis patients [28,29], the FR-β may be an interesting target for imaging of inflammatory diseases [30].

In the last two decades a variety of folate conjugates for nuclear imaging via PET and SPECT have been developed [6,31,32]. Only two of these folate-based radioconjugates—^111^In-DTPA-folate and ^99m^Tc-EC20—exist which have been tested in clinical trials in human patients [33,34]. ^99m^Tc-EC20 (Etarfolatide^TM^, Endocyte Inc.) is currently being employed in several clinical trials for the selection of patients who may profit from FR-targeted therapies [35]. Regarding folate-based radiotracers for PET imaging, none of them has been tested in clinical trials yet.

**Figure 1:**
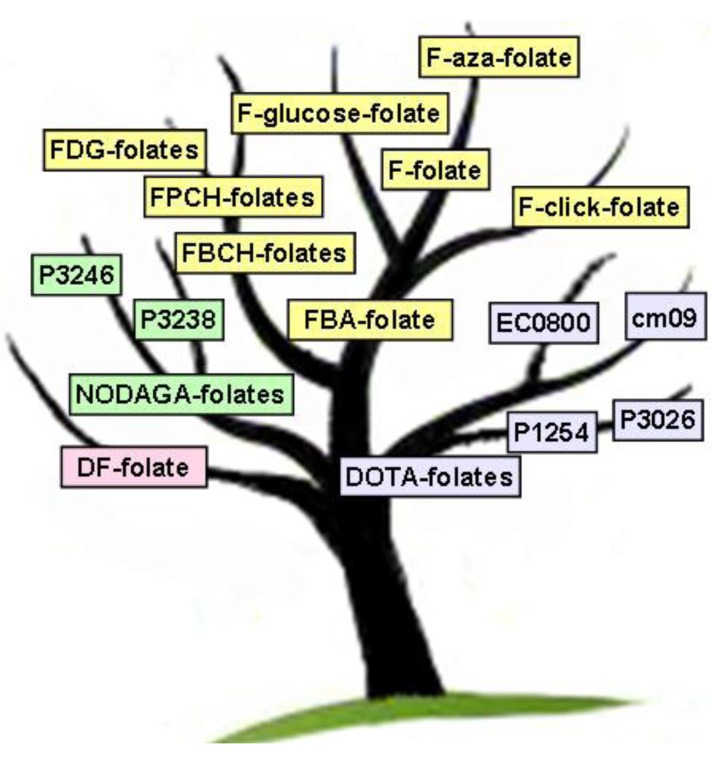
“Folate-Tree”—evolution of folate-based PET agents for ^18^F-labeling (yellow) and for radiometallation using variable chelators (pink: DF = deferoxamine; green: NODAGA = 1,4,7-triazacyclononane,1-glutaric acid-4,7-acetic acid; blue: DOTA = 1,4,7,10-tetraazacyclododecane-*N,N',N'',N'''*-tetraacetic acid) suitable for radiolabeling with ^66/68^Ga, ^44^Sc or ^152^Tb.

However, extensive research has been dedicated to the development and pre-clinical evaluation of folate radiotracers for PET. An overview of the evolution of these folate-based PET agents is given in Figure 1. In the following chapters we summarize and discuss the development of these folate radiotracers structured according to the PET isotopes ^18^F, ^68/66^Ga, ^152^Tb and ^44^Sc which have been employed.

For evaluation of the large majority of these folate-based PET tracers, KB tumor xenografted athymic nude mice have been used. The human cervical KB cancer cell line is a subclone of HeLa cells [36], known to express the FR-α at high levels. Hence, KB cells became the standard cell line for testing folic acid radioconjugates *in vitro* and in tumor xenografted athymic nude mice.

## 2. PET Isotopes Used for the Development of Folate Radiotracers

The PET isotopes which have been used in conjunction with folate conjugates are shown in Table 1. Among those are radioisotopes which are established in current clinical practice (e.g., ^18^F, ^68^Ga), but also novel isotopes which are under development for potential clinical use in the future (e.g., ^44^Sc).

^18^F is the most widely used radionuclide for PET imaging because of its favorable decay characteristics (Table 1) [37]. The short positron range as a consequence of the low positron energy of ^18^F is responsible for an excellent PET image resolution which is superior to the resolution achieved with other currently available positron emitters [37]. Another advantage of ^18^F-based radiotracers may be the minimal structural alteration of fluorinated tracers compared with radiometallation that requires derivatization of the molecule with a suitable chelating agent. These characteristics make the development of an ^18^F-radiolabeled folate-based radiotracer particularly appealing for clinical PET applications.

Clinical application of ^68^Ga in conjunction with targeting agents (e.g., somatostatin analogs) proved the favorable characteristics of this radioisotope [5,38]. PET imaging of malignancies with high resolution and excellent sensitivity allows quantification of tracer uptake within smallest lesions. The reason why ^68^Ga has become a relevant isotope for routine use in the clinics lies also in its easy availability by a ^68^Ge/^68^Ga-generator which makes its use independent of an on-site cyclotron in hospitals [39,40]. However, there are drawbacks such as the short half-life (t_1/2_ = 68 min, Table 1) and the fact that there is still no generator commercially available which is approved by the authorities. This might hinder future application of this radioisotope.

^66^Ga is an isotope with decay properties potentially suitable for PET imaging (Table 1). However, the lower abundance of positrons and the more complicated spectrum of γ-rays that are emitted in cascade did not make ^66^Ga widely accepted for application in radiopharmaceutical science [41]. 

**Table 1:** PET isotopes employed in conjunction with folate derivatives—physical decay properties and production routes.

Isotope	Half-life	Energy_av_ β^+^ [keV]	Intensity [%]	Production Method	Folates Ref.
^18^F	110 min	250	97	cyclotron ^18^O(p,n)^18^F	[42,43,44,45,46]
^66^Ga	9.5 h	1750	57	cyclotron ^66^Zn(p,n)^66^Ga	[47]
^68^Ga	68 min	830	89	^68^Ge/^68^Ga-generator	[48,49,50,51]
^152^Tb	17.5 h	1080	17	high-energy proton-induced spallation of tantalum targets (ISOLDE/CERN)	[52]
^44^Sc	3.97 h	632	94	(i) ^44^Ti/^44^Sc-generator (ii) cyclotron ^44^Ca(p,n)^44^Sc	[53]

Terbium is one of the few lanthanides which comprises several clinically interesting radioisotopes, among those the positron-emitting ^152^Tb (Table 1). However, the production of ^152^Tb is more difficult than it is the case for other PET isotopes. ^152^Tb can be produced by high-energy proton induced spallation of tantalum targets followed by an on-line separation process, e.g., at a site like the CERN Isotope Separator On Line (ISOLDE, Geneva, Switzerland) [54,55]. This was the method applied for production of ^152^Tb used for radiolabeling of a folate conjugate as reported below [52]. ^152^Tb is stably coordinated by the established macrocyclic chelator DOTA which is employed also for the therapeutic radioisotopes ^177^Lu and ^90^Y and hence, it may be applied as a “matched pair” with these therapeutic radioisotopes. The relatively long half-life of ^152^Tb may cover imaging periods over several hours or even days. These features would make ^152^Tb attractive for patient specific dosimetry and therapy monitoring by using PET. However, due to the limited availability of this radioisotope pre-clinical *in vivo* studies using ^152^Tb-radiolabeled biomolecules are still scarce.

^44^Sc decays by emission of positrons with a half-life of 3.97 h (Table 1). It may be particularly useful as a diagnostic match for the therapeutic isotope ^47^Sc (β^−^-emitter, t_1/2_ = 3.35 d) and possibly also for ^177^Lu (β^−^-emitter, t_1/2_ = 6.65 d) [56]. So far, there are only few pre-clinical PET studies performed with ^44^Sc-labeled biomolecules [57,58]. Production of ^44^Sc is accessible through a ^44^Ti/^44^Sc generator system [59] or via a (p,n)-nuclear reaction by irradiation of ^44^Ca targets at a cyclotron (Table 1) [60]. Due to the almost 4-fold longer half-life of ^44^Sc compared to ^68^Ga it would be possible to deliver ^44^Sc-based radiopharmaceuticals to hospitals located several hundred kilometers far from the production facility. For the studies performed with a DOTA-folate conjugate reported below, ^44^Sc was produced at the cyclotron at the Paul Scherrer Institute (Villigen-PSI, Switzerland) by irradiation of enriched ^44^Ca targets [53].

## 3. Design and Application of [^18^F]Fluorofolate Radiotracers

### 3.1. General Design of ^18^F-Folate Radiotracers

In recent years, several ^18^F-labeled folate derivatives have been developed and evaluated in pre-clinical studies [6]. These derivatives can be subdivided in two groups of tracer designs. The first design of ^18^F-folate derivatives was based on a ^18^F-labeled prosthetic group which was conjugated to a folate precursor [42,43,45]. This approach is called “pendent approach” and represents a more conventional method for the preparation of folate radiotracers. A major drawback of this approach was the complex and usually time-consuming multistep radiosynthesis, which would make the translation to an automated system difficult [46]. A second group of radiotracers were designed according to the “integrated approach”, where the ^18^F-label was directly attached to the folate molecule’s backbone [44,46]. The advantage of the “integrated approach” was the simple and quick radiosynthesis of the folate tracer which would open the possibility for routine production on a modular system for potential clinical application [46].

### 3.2. [^18^F]Fluoro-Benzylamine-Folate

**Figure 2:**
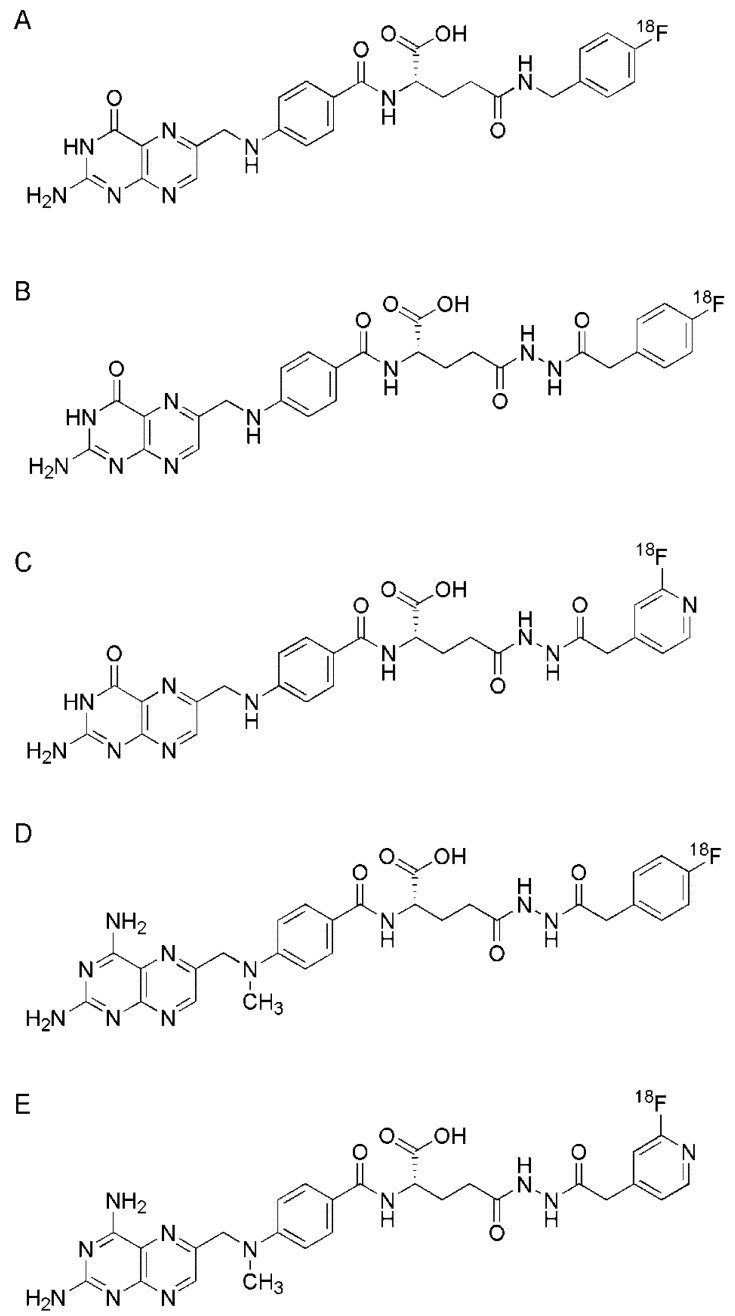
(**A**) Chemical structures of [^18^F]FBA-γ-folate [63], (**B**) [^18^F]fluoro-benzene-carbohydrazide-folates, (**C**) [^18^F]fluoro-pyridine-carbohydrazide-folate [63], (**D**) [^18^F]fluoro-pyridine-carbohydrazide-methotrexate [67] and (**E**) [^18^F]fluoro-pyridine-carbohydrazide-methotrexate [67].

Bettio *et al.* were the first who reported on the development and *in vivo* application of a ^18^F-based folate radiotracer [42]. Radiosynthesis of the prosthetic group was performed by radiofluorination of 4-cyano-*N,N,N*-trimethylanilinium trifluoromethanesulfonate as previousely reported [61] followed by purification over a C-18 Sep-Pak cartridge and reduction of the nitrile functionality. After isolation of the fluorinated prosthetic group containing a free amino-group the coupling reaction was carried out with in-situ activated un-protected folic acid. This reaction step including HPLC purification yielded the final product, an isomeric mixture of [^18^F]fluorobenzylamine-α/γ-folic acid ([^18^F]FBA-α/γ-folate, Figure 2A), by 15%–44% at a specific activity of up to 24 GBq/μmol. An HPLC system with an acidic eluent (pH 3.5) allowed discrimination among the α- and γ-isomers, which were formed at a ratio of 1:4, as confirmed by HPLC co-injection of the corresponding non-radioactive reference compounds [42]. *In vitro* testing revealed binding affinities in the same range for both the α-and γ-isomer, which justified the *in vivo* use of the isomeric mixture of this radiotracer. PET images were acquired using a dedicated small-animal PET scanner (quad-HIDAC tomograph, Oxford Positron Systems, Weston-on-the-Green, Oxfordshire, UK [62]). In a first step, mice bearing KB tumor xenografts were scanned 30 min after injection of [^18^F]FDG (16.9 MBq). Two days later additional PET imaging studies were performed with the same mice 75 min after injection of [^18^F]FBA-α/γ-folate (13.3 MBq) [42]. Tumor uptake of radioactivity was observed upon injection of the folate radiotracer whereas accumulation of [^18^F]FDG was largely absent. The excellent resolution of the PET images allowed even visualization of heterogeneous uptake of [^18^F]FBA-α/γ-folate within the tumor xenograft. In healthy organs and tissue highest retention of radioactivity was seen in the liver and in the kidneys [42]. *Ex vivo* biodistribution studies confirmed FR-specific uptake in KB tumor xenografts and in the kidneys since co-injection of excess folic acid to block the receptors resulted in a significant decline of radiotracer accumulation.

### 3.3. [^18^F]Fluoro-Benzene- and [^18^F]Fluoro-Pyridinecarbohydrazide-Folates

Al Jammaz *et al*. reported the synthesis of [^18^F]fluorobenzene- and [^18^F]fluoropyridinecarbohydrazide-folates (Figure 2B,C) [63]. In the first approach the prosthetic groups 4-[^18^F]fluorobenzoate and 2-[^18^F]fluoro-4-pyridinecarboxylate [64] were reacted with hydrazine hydrate to give the corresponding 4-[^18^F]fluorobenzenecarbohydrazide and 2-[^18^F]fluoropyridine-4-carbohydrazide for subsequent conjugation with NHS-activated folic acid. The folate conjugates were purified using Sep-Pak silica cartridges. The overall radiochemical yields were > 80% for both conjugates with a total synthesis time of 45 min. In the second approach 4-[^18^F]fluorbenzoate was converted to the corresponding acid for reaction with hydrazide-γ-folate which was synthesized as previously reported and separated from the α-isomer by HPLC [65,66]. After purification using a Sep-Pak silica column, the fluorinated folate conjugate was obtained in an overall yield of 35% and after a total synthesis time of 85 min [63].

In addition, further ^18^F-conjugates were synthesized according to the same radiosynthetic strategy [63], but instead of using folic acid as the targeting ligand the ^18^F-labeled prosthetic groups was conjugated to the antifolate methotrexate (Figure 2D,E) [67]. Methotrexate is a clinically well-established chemotherapeutic agent that may enter tumor cells partially by FRs although its primary route is mediated via the reduced folate carrier (RFC) [68]. Moreover, the [^18^F]fluorobenzene- and [^18^F]fluoropyridinecarbohydrazide folate and methotrexate conjugates were tested *in vitro* using KB tumor cells in culture and *in vivo* using KB tumor bearing mice [67]. Cell binding revealed similar binding affinities of the two fluoro-folates whereas in the case of the methotrexate-based compounds a two-fold lower binding affinity was observed [67].

*In vivo* tissue distribution studies in KB tumor bearing mice revealed clearly better results for the [^18^F]fluoropyridine-carbohydrazide-folate compared to [^18^F]fluorobenzene-carbohydrazide-folate with regard to a reduced uptake in non-targeted tissues and organs [67]. These findings may be attributed to the more hydrophilic character of the [^18^F]fluorpyridine-carbohydrazide prosthetic group compared to the [^18^F]fluorobenzene-based prosthetic group. The same tendency was also observed for the two methotrexate conjugates [67]. However, accumulation of the methotrexate-based radiotracers in tumors was low compared to the folate conjugates which showed an >6-fold higher tumor uptake [67]. *In vivo* PET (YAPPET scanner, ISE, Pisa, Italy) was performed with anesthetized mice 45 min after injection of [^18^F]fluoropyridine-carbohydrazide-folate (11–18.5 MBq) allowing visualization of the accumulated radioactivity in the tumor xenografts and in the kidneys [67].

### 3.4. [^18^F]Fluoro-Click-Folate

**Figure 3:**
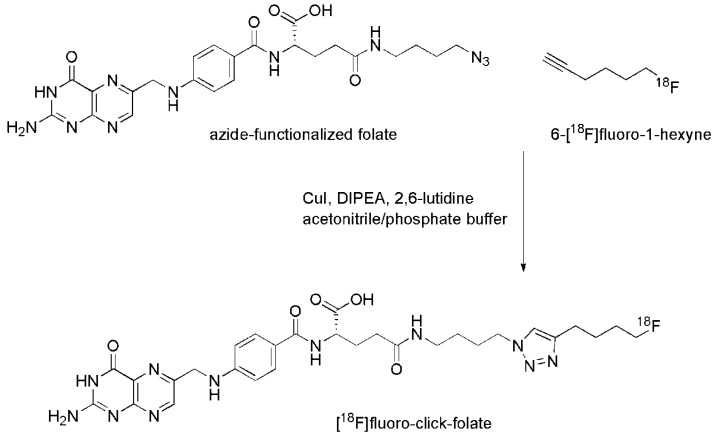
Radiosynthesis of [^18^F]fluoro-click-folate [43] using an azide functionalized folate precursor and 6-[^18^F]fluoro-1-hexyne as a prosthetic group. (DIPEA = *N,N*-diisopropylethylamine).

Ross *et al.*, developed a folic acid conjugate in which 6-[^18^F]fluoro-1-hexyne was used as a radioactive prosthetic group for coupling via the Cu(I)-catalyzed 1,3-dipolar cycloaddition [69,70] to an azide-derivatized folate precursor (Figure 3) [43]. 6-[^18^F]fluoro-1-hexyne was synthesized starting from the corresponding *p*-tosylate precursor according to a previously published procedure [71].

In a second step 6-[^18^F]fluoro-1-hexyne was reacted with γ-(4-azidobutyl)-folic acid amide [72] in the presence of Cu(I) as a catalyst to form the 1,4-triazole moiety [43]. Stirring the solution at elevated temperature resulted in a conversion of 65–80% of the [^18^F]fluoro-click-folate (Figure 3). After purification using HPLC, the solvents were evaporated and the final product was formulated in PBS suitable for biological applications [43]. Cell binding studies of the non-radioactive reference compound revealed a somewhat lower FR-affinity than what was found with native folic acid. *In vivo* application showed a moderate uptake in KB tumor xenografts (3.13 ± 0.83% ID/g 45 min p.i.) which was, however, blockable by pre-injection of excess folic acid (0.19 ± 0.08% ID/g 45 min p.i.) [43]. Whereas retention in the kidneys was comparatively low, high uptake of this novel ^18^F-folate tracer was found in the bile and feces indicating its hepatobiliar exrection, probably as a consequence of an increased hydrophobicity of this folate radioconjugate compared to [^18^F]FBA-α/γ-folate [42]. Whole-body PET images of KB tumor bearing mice were acquired with a dedicated small-animal PET scanner (Oxford Positron Systems quad-HIDAC tomograph [62]). The images showed highest accumulation of radioactivity in the excretory organs such as the gall bladder, intestines, urinary bladder and kidneys as it was expected from *post mortem* data [43]. Tumor xenografts could be visualized, but solely on those PET images which represented scans of the region of the head and thorax and not on whole-body scans where radioactive uptake was dominating in the abdominal region [43,73].

### 3.5. [^18^F]Fluoro-Glucose-Folates

**Figure 4:**
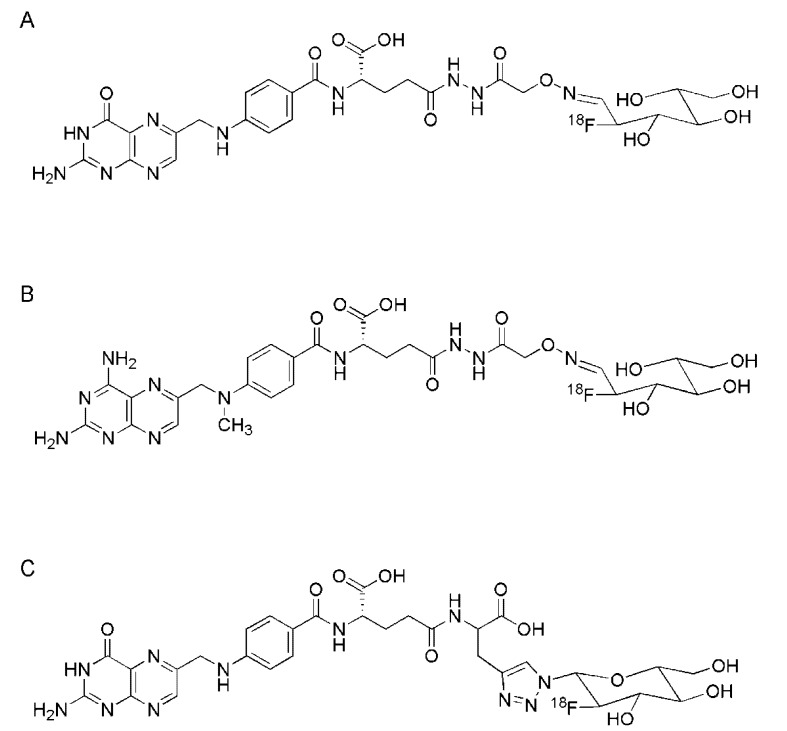
(**A**) Chemical structures of [^18^F]FDG-folate [74], (**B**) [^18^F]FDG-methotrexate [74] and (**C**) [^18^F]fluoro-deoxy-glucose-folate [45].

In an attempt to design a more hydrophilic folate-based ^18^F-radiotracer, Al Jammaz *et al*. reported the synthesis and evaluation of conjugates with [^18^F]FDG as a prostethic group (Figure 4A,B) [74]. The folate- and methotrexate-carbohydrazide derivatives were synthesized as previously reported [63,67]. Then, they were reacted with aminoxyacetyl chloride to yield aminoxy-functionalized folate- and methotrexate derivatives used for the coupling reaction with [^18^F]FDG [74]. For this purpose, [^18^F]FDG as oxime-forming prosthetic labeling reagent was synthesized and reacted with the folate or methotrexate derivatives. The reaction was accomplished at 60 °C within 10–15 min followed by purification using a silica Sep-Pak column. Quality control revealed a radiochemical purity of >98%. The overall radiochemical yield was >80% at specific activities of >9 GBq/μmol and a total synthesis time of only about 20 min [74]. *In vitro* both of the radiotracers [^18^F]FDG-folate (Figure 4A) and [^18^F]FDG-methotrexate (Figure 4B) were sufficiently stable in human plasma over 4 h.

The *in vivo* tissue distribution data obtained from KB tumor bearing mice showed moderate tumor uptake (3.32 ± 0.32% ID/g, 60 min p.i.) of the folate conjugate and very low accumulation in the kidneys (1.49 ± 0.05% ID/g, 60 min p.i.). Again, these findings were different from previous findings with folate radioconjugates which always showed low tumor-to-kidney ratios [6,31]. Al Jammaz *et al*. stated that this behavior may be attributed to the nature of [^18^F]FDG as a prosthetic group and the overall negative charge of the folate conjugate [74]. Accumulation of the methotrexate conjugate in tumors was about 3-fold lower and retention in the kidneys no higher than background [67,75]. The authors concluded the study by mentioning the convenient one-step radiosynthesis, high yield and short synthesis time which would make the [^18^F]FDG-folate conjugate suitable for large-scale production [74].

A smart approach for the preparation of a [^18^F]fluoro-deoxy-glucose-based folate radiotracer (Figure 4C) was recently reported by Fischer *et al*. [45]. The aim of this study was to combine the advantage of the “click-chemistry” approach for the coupling reaction of a ^18^F-labeled prosthetic group to folic acid [43] with the employment of a hydrophilic prosthetic group of a well-established radiolabeling procedure. By application of the concept previously introduced by Maschauer *et al*. [76,77] an azide-functionalized [^18^F]fluorinated glucose entity was conjugated by the Cu(I)-catalyzed “click-reaction” with an alkyne-derivative of folic acid [45]. For the preparation of the folate-alkyne a protected pteroic acid precursor was reacted with an alkyne functionalized glutamic acid intermediate protected at the α-carboxylate group according to a previously published procedure [72]. Upon deprotection of the folate-alkyne derivative it was purified using a C-18 reversed-phase cartridge. The preparation of the protected glucose-azide was obtained by nucleophilic ^18^F-substitution of the mannosyl-precursor, followed by purification using an C-18 reversed-phase cartridge as previously reported [76]. Upon hydrolysis and neutralization of the crude ^18^F-labeled glucose-based prosthetic group it was directly used for the “click-reaction” which was accomplished at 50 °C within 15 min. After purification of the final product using HPLC, [^18^F]fluoro-deoxy-glucose-folate conjugate (Figure 4C), was obtained at a radiochemical yield of 5–25% and a radiochemical purity of >95% at a maximal activity amount of 1–3 GBq. *In vitro* the fluoro-deoxyglucose-folate reference compound bound specifically to FRs of KB cells with an affinity in the same range as native folic acid [45]. The logD value (−4.2 ± 0.1) indicated very hydrophilic characteristics and stability experiments in human blood plasma showed no defluorination of [^18^F]fluoro-deoxy-glucose-folate over at least 2 h. In biodistribution studies performed in nude mice [^18^F]fluoro-deoxy-glucose-folate showed high and FR-specific accumulation in KB tumor xenografts (10.03 ± 1.12% ID/g, 60 min p.i.). Significant retention was also observed in other FR-positive organs and tissue such as the kidneys (42.94 ± 2.04% ID/g, 60 min p.i.) and salivary glands (5.93 ± 0.77% ID/g, 60 min p.i.) [45]. Retention of radioactivity in the bile and feces was much lower compared to the previously evaluated [^18^F]fluoro-benzylamine-folate [42] and [^18^F]fluoro-click-folate [43]. The reason for the relatively high liver uptake compared to other ^18^F-based folate radiotracers remained unclear [45].

**Figure 5:**
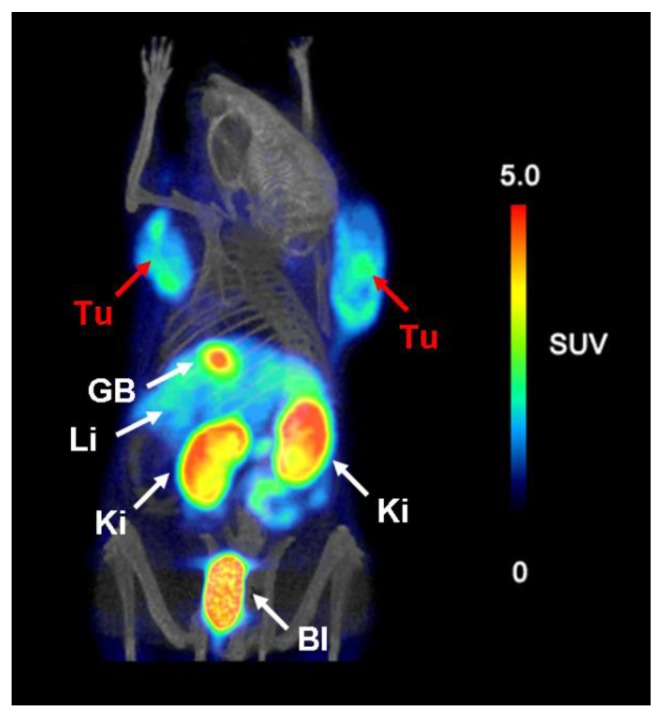
PET/CT image of a mouse 75 min after injection of [^18^F]fluoro-deoxy-glucose-folate (~14 MBq) [45]. (Tu = KB tumor xenograft, GB = gall bladder, Li = liver, Ki = kidney, Bl = urinary bladder). Reprinted (adapted) with permission from Fischer *et al*. [45] Copyright (2012) American Chemical Society.

Images of mice were obtained 75 min after injection of ~14 MBq [^18^F]fluoro-deoxy-glucose-folate with a dedicated small-animal PET/CT camera (eXplore VISTA, GE Healthcare, Waukesha, WI, USA). Accumulation of radioactivity was found in KB tumor xenografts located on each shoulder (Figure 5) [45]. The same hold true for the kidneys. At both sites accumulation of radioactivity was reduced significantly in a mouse co-injected with excess folic acid whereas uptake in the gall bladder, liver and urinary bladder were not reduced under blockade conditions [45]. Among current folate-based ^18^F-tracers, [^18^F]fluoro-deoxy-glucose-folate is one of the most promising radiotracers with regard to the high-yielding radiosynthesis and excellent *in vivo* tissue distribution data.

### 3.6. Integrated Approach: 2'-[^18^F]Fluorofolic Acid and 3'-Aza-2'-[^18^F]Fluorofolic Acid

**Figure 6:**
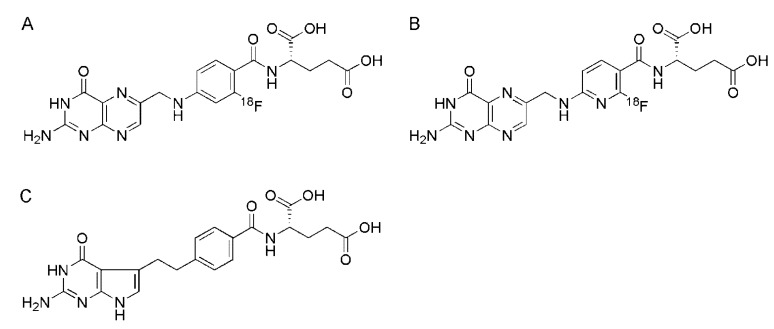
(**A**) Chemical structures of 2'-[^18^F]fluorofolic acid, (**B**) 3'-aza-2'-[^18^F]fluorofolic acid and (**C**) pemetrexed.

While pursuing the development of folate-based ^18^F-radiotracers potentially suitable for routine production, Ametamey and co-workers reported on a new concept where the ^18^F-label was integrated into the folic acid backbone instead of using a radiolabeled prosthetic group [44]. Aromatic substitution of a leaving group at the 2'-position of the 4-amino-benzoyl moiety of folic acid appeared to be an appealing option for direct ^18^F-labeling of folic acid. For the preparation of 2'-[^18^F]fluorofolic acid (Figure 6A) a protected folate precursor (*N^2^*-(*N,N*-dimethylaminomethylene)-2'-nitrofolic acid *di*-*tert*-butylester [78]) was labeled with ^18^F via a direct nucleophilic aromatic substitution of the 2'-nitro group at 140 °C [44].

The final radiotracer was achieved with a radiochemical purity which was always >95%. The maximal overall decay-corrected yield was about 4% and the synthesis time 80 min [44]. Binding affinity tests using the non-radioactive reference compound revealed high-affine FR-binding comparable to native folic acid. Tissue distribution studies performed in KB tumor bearing nude mice showed high and specific accumulation of the radiotracer in tumor xenografts (9.37 ± 1.76% ID/g, 75 min p.i.). Expectedly, FR-specific uptake was found in the kidneys whereas accumulation of the radiotracer in the bile and feces were relatively low. 2'-[^18^F]Fluorofolic acid was also tested in combination with pre-injected pemetrexed. Pemetrexed is a multitargeted antifolate which inhibits many folate-dependent reactions that are essential for cell proliferation [79]. It is used in the clinic in combination with cisplatin for the treatment of non-small cell lung cancer and mesothelioma [79,80]. In our previous research studies pemetrexed showed a favorable effect on the tissue distribution of folate-based radiotracers [81,82,83,84].

After administration of pemetrexed accumulation of 2'-[^18^F]fluorofolic acid was largely maintained in the tumor tissue whereas the unfavorably high uptake in the kidneys was reduced from 35.73 ± 0.25% ID/g to 14.05 ± 1.02% ID/g at 75 min p.i. [44]. In addition the uptake in the liver and feces was also reduced [44]. PET imaging studies were performed with tumor bearing mice 75 min after injection of 2'-[^18^F]fluorofolic acid. KB tumor xenografts and kidneys were clearly visualized. The uptake in these tissue and organs was reduced in mice which received excess folic acid [44].

This study exemplified the feasibility of using folate derivatives of the “integrated approach” with only minimal structural alteration of native folic acid as effective tumor targeted radiotracers. A drawback was however, the low yield of the radiosynthesis which would not have been suitable for production of this radiotracer on a modular system. Nevertheless, at the time 2'-[^18^F]fluorofolic acid clearly outperformed any previous ^18^F-based radioconjugate with regard to the *in vivo* tissue distribution characteristics [44].

More recently, Betzel *et al*. presented a novel folate based radiotracer which was designed according to the “integrated approach” [46]. To overcome the problem of a low radiochemical yield which was experienced during the production of 2'-[^18^F]fluorofolic acid [44], the phenyl ring of folic acid was isosterically replaced by a pyridine moiety resulting in an aza-folic acid derivative. It was reasoned that nucleophilic aromatic [^18^F]fluorination at the 2'-position of the pyridine ring in 3'-aza-folic acid would result in a higher radiochemical yield. To produce a 3'-aza-2'-[^18^F]fluorofolic acid (Figure 6B) the folate precursor *N^2^*-acetyl-3'-aza-2'-chlorofolic acid di-*tert*-butylester was used [46].

**Figure 7:**
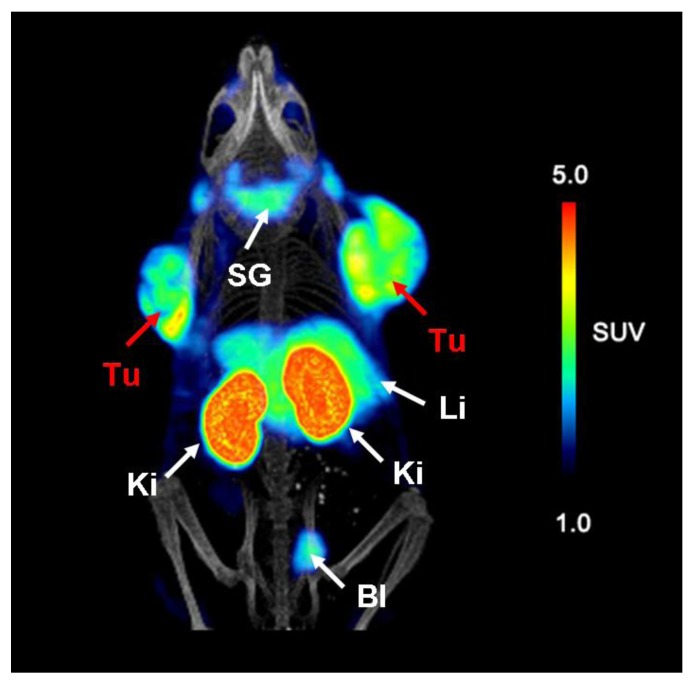
(**a**) PET/CT image of a tumor bearing mouse 2 h after injection of 3'-aza-2'-[^18^F]fluorofolic acid (~29 MBq) [46]. Reprinted (adapted) with permission from Betzel *et al*. [46] Copyright (2013) American Chemical Society.

The radiosynthesis was carried out in two steps. Firstly, the chloride leaving group was replaced by [^18^F]fluoride within 10 min at 160 °C and secondly the radiolabeled compound was deprotected under acidic conditions at 60 °C within another 10 min. Upon purification using semipreparative HPLC, 3'-aza-2'-[^18^F]fluorofolic acid was obtained in an overall yield of 3–9% with a radiochemical purity of > 98% and a specific activity of up to 127 GBq/μmol. The total synthesis time was 110 min [46]. The IC_50_-value indicating FR-binding affinity was in the same range as previously found for the 2'-fluorofolic acid [44]. In addition, cell internalization studies performed with 3'-aza-2'-[^18^F]fluorofolic acid showed FR-specific uptake and the logD value indicated a very hydrophilic character. *In vivo* a high uptake of the radiotracer was observed in KB tumor xenografts (11.70 ± 0.87% ID/g, 30 min p.i.) already short after administration [46]. Accumulation in the kidneys was in the range of 53–58% ID/g over the time of investigation from 30 min to 90 min p.i. resulting in tumor-to-kidney ratios of around 0.2. Besides, FR-specific uptake was only found in the salivary glands. PET/CT imaging studies were performed with KB tumor bearing nude mice using a dedicated small-animal PET/CT scanner (eXplore VISTA). On PET/CT images taken 2 h after injection of ~29 MBq of 3'-aza-2'-[^18^F]fluorofolic acid tumor visualization was excellent and undesired accumulation of radioactivity was found only in the kidneys, salivary glands and in the liver (Figure 7) [46]. The authors concluded that the new 3'-aza-2'-[^18^F]fluorofolic acid radiotracer may serve as an appropriate diagnostic tool for imaging FR-positive diseased tissue. The fast and easy radiosynthesis would be a major advantage for translation of the radiosynthesis to an automated synthesis module allowing application of this novel folate radiotracer in a clinical study [46].

## 4. Folate Conjugates for Radiolabeling with [^66/67/68^Ga]Gallium

### 4.1. Deferoxamine-Folate

One of the earliest reports on folic acid radioconjugates was about the synthesis and application of a ^67^Ga-deferoxamine folate by Mathias *et al*. in the late 1990ies [48,49]. A few years later, the same deferoxamine-folate conjugate was radiolabeled with the two positron emitting gallium isotopes, cyclotron-produced ^66^Ga and generator-produced ^68^Ga (Table 1) [47]. Radiolabeling with both radioisotopes was performed by incubation of a mixture of the deferoxamine-folate conjugate and a solution of radioactive gallium in acetylacetone/ethanol at 50 °C for 15–30 min. This resulted in a specific activity of 18 MBq/μg and a radiochemical yield of >97%. MicroPET imaging experiments were performed in KB tumor bearing mice 25 h after injection of ^66^Ga-deferoxamine-folate (174 MBq/11 μg) using a small-animal PET scanner (Concorde Microsystems microPET R4, Knoxville, TN, USA) [47]. Magnetic resonance imaging was performed to confirm anatomic correlation with the mircoPET results. The FR-positive tumors and the kidneys were readily visualized and co-injection with folic acid resulted in the expected reduction of renal and tumor accumulation of ^66^Ga-deferoxamine-folate. However, very high accumulation of radioactivity was found in the abdominal region of mice [47] as expected from *post mortem* data previously obtained with ^67^Ga-deferoxamine folate [49]. The authors highlighted the feasibility of microPET tumor imaging with ^66^Ga in spite of its much higher positron energy (E_av_β^+^: 1750 keV) compared to ^68^Ga and ^18^F (Table 1) [47].

### 4.2. DOTA-Folates and DO3A-Pteroate

**Figure 8:**
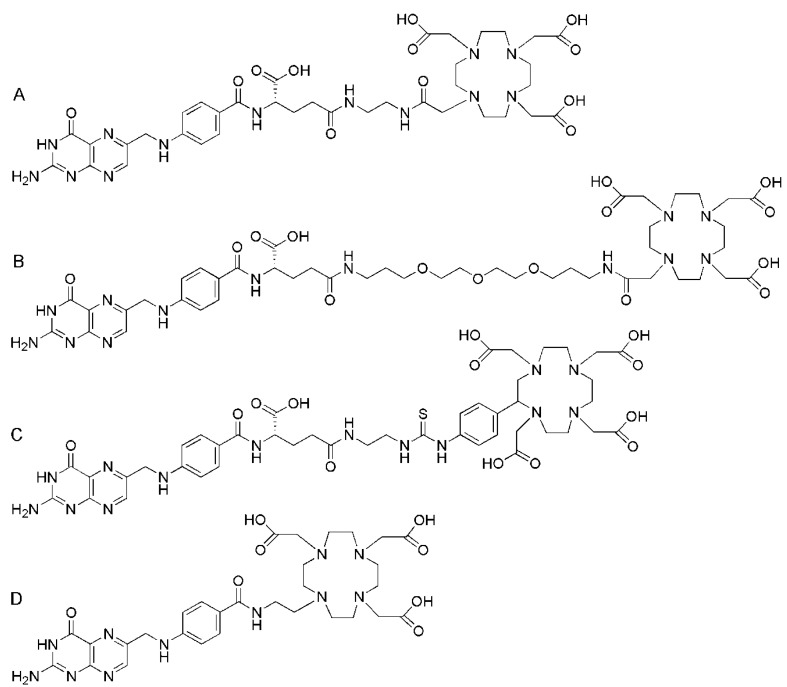
(**A**) Chemical structures of the DOTA-folate P3026 [50], (**B**) the DOTA-folate P1254 which comprises a short PEG spacer [50], (**C**) EC0800 (Endocyte Inc., West Lafayette, IN, USA) [85] and (**D**) DO3A-pteroate [86].

Three different DOTA-folate conjugates were synthesized and evaluated for labeling with ^67/68^Ga [50,85]. Fani *et al*. reported on the development of a DOTA-folate conjugates with a 1,2-diaminoethane linker, referred to as P3026 (Figure 8A) and a DOTA-folate conjugate with a short PEG spacer (*i.e.*, 3-{2-[2-(3-aminopropoxy)-ethoxy]-ethoxy}-propylamine), referred to as P1254 (Figure 8B) [50].

*In vitro* both radioconjugates ^67/68^Ga-P3026 and ^67/68^Ga-P1254 showed an increased KB tumor cell uptake and higher retention over time compared to ^111^In-DTPA-folate, which was prepared for comparative studies as it was the first folate radioconjugate applied in a clinical trial [33]. The *post mortem* data obtained with the two ^67/68^Ga-DOTA-folate conjugates showed largely the same results. The tumor uptake was about 10% ID/g at 2 h p.i., whereas accumulation in the kidneys differed slightly among the two radiotracers (^67/68^Ga-P3026: 87.78 ± 12.37% ID/g; ^67/68^Ga-P1254: 98.43 ± 15.40% ID/g) [50]. Tumor-to-kidney ratios were between 0.08 and 0.14 for the entire time interval of investigation (20 min to 24 h p.i.). PET experiments were performed with tumor bearing mice 1 h after injection of 4 MBq ^68^Ga-P3026 (0.4 nmol) using a routine PET/CT scanner (Discovery STE, GE Medical Systems, Waukesha, WI, USA). Before imaging mice were sacrificed and the kidneys were surgically removed in order to allow localization of the tumor xenografts without disturbance from high radioactivity accumulated in the renal tissue [50].

Müller *et al*. investigated a DOTA-Bz-EDA-folate conjugate (referred to as EC0800, Figure 8C) which was developed by Endocyte Inc. [85]. All of the experiments reported in this article were performed with the ^67^Ga-labeled version of EC0800. The evaluation in KB tumor bearing nude mice revealed high accumulation of ^67^Ga-EC0800 in tumor xenografts (6.08 ± 0.89% ID/g, 4 h p.i.). Besides, only FR-positive tissues such as the salivary glands (6.93 ± 1.67% ID/g) and the kidneys (84.53 ± 14.10% ID/g) accumulated significant amounts of radioactivity [85]. In an unpublished study, EC0800 was also labeled with ^68^Ga for PET imaging. Two KB tumor bearing mice were scanned 1.5 h after injection of ^68^Ga-EC0800 with and without pre-injeced pemetrexed using a small-animal PET/CT scanner (eXplore VISTA, GE Healthcare).

**Figure 9:**
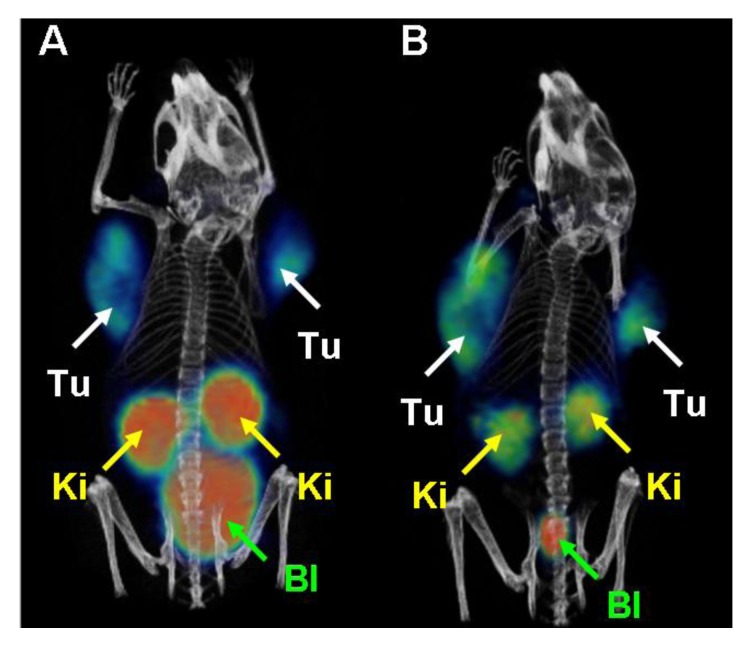
PET/CT images of KB tumor bearing mice. (**A**) Mouse injected with ^68^Ga-EC0800 (~25 MBq) 1.5 h before scanning and (**B**) mouse which received pemetrexed (0.4 mg) prior to ^68^Ga-EC0800 (~18 MBq). (Tu = KB tumor xenograft, Ki = kidney, Bl = urinary bladder). Reprinted (adapted) with permission from Müller *et al*. [6]. Copyright (2013) Bentham Science Publishers.

In both mice accumulation of radioactivity was seen in tumors, kidneys and in the urinary bladder (Figure 9) [6]. In contrast to the control mouse where kidneys showed very high uptake of ^68^Ga-EC0800 (Figure 9A), a clearly improved tumor-to-kidney ratio of almost one was observed in the mouse which received pemetrexed prior to the radiotracer (Figure 9B).

Omitting the glutamate moiety of folic acid (pteroyl-glutamic acid) may be a measure to circumvent the challenge of the preparation of chemically pure α- or γ-isomers of folate conjugates. It has been shown in previous experiments with pteroate-based radiotracers that the glutamate moiety is not essential to maintain FR-targeting [87,88,89]. Kühle *et al*. reported on the organic synthesis of a pteroate derivative with a DO3A-chelator (Figure 8D) [86]. Radiolabeling of the DO3A-pteroate (30 nmol) was performed in HEPES buffer (0.13 M) at 95 °C with a radiochemical yield of 75% after 10 min [86]. The ^68^Ga-DO3A-pteroate was stable in PBS over at least 3 h even in the presence of excessive amounts of transferrin. The distribution coefficient revealed a logD value of −0.1 ± 0.1 [86]. In the literature, it is reported that a spacer between the pteroate moiety and the imaging or therapeutic probe would be advantageous to maintain FR-targeting properties of pteroate conjugates [90,91]. Future experiments with the novel ^68^Ga-DO3A-pteroate in tumor bearing mice may provide conclusive results to finally answer the question about a potential need of a spacer entity for pteroate conjugates.

### 4.3. NODAGA-Folates

**Figure 10:**
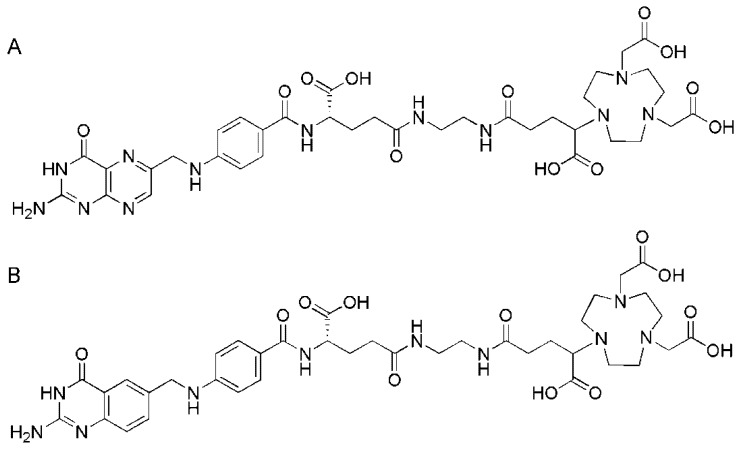
(**A**) Chemical structure of the NODAGA-folate P3246 [51] and (**B**) the NODAGA-dideaza-folate P3238 [51].

Recently, preparation and biological evaluation of two NODAGA-folate conjugates for FR-targeted cancer imaging have been reported by Fani *et al.* [51]. One of the derivatives, referred to as P3246, was a conventional folic acid conjugate which was linked to the NODAGA-chelator via a 1,2-diaminoethane spacer (Figure 10A).

The second derivative, referred to as P3238, was based on a 5,8-dideazafolic acid skeleton known from the chemical structure of the antifolate CB3717 [92] which was linked to the same chelator/linker entity (Figure 10B) [51]. Radiolabeling with ^68^Ga was carried out in 2 mL sodium acetate buffer pH 4.0 at room temperature within 10 min. ^68^Ga-P3246 and ^68^Ga-P3238 were obtained at a specific activity of 30 MBq/nmol and a labeling yield of >95%.

**Figure 11:**
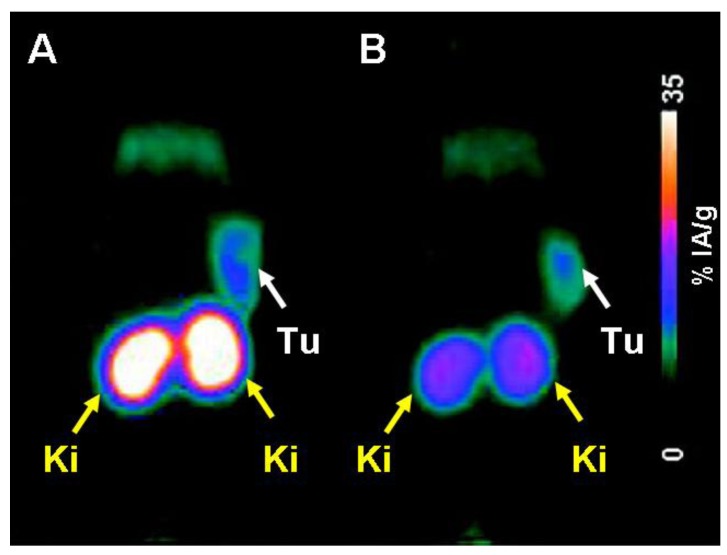
MIP PET images of ^68^Ga-P3246 1 h p.i. without (**A**) and with pre-injection of pemetrexed (**B**). (Tu = KB tumor xenograft, Ki = kidney) [51]. Reprinted (adapted) with permission from Fani *et al*. [51]. Copyright (2012) American Chemical Society.

*In vitro* and *in vivo* evaluation of ^68^Ga-P3246 and ^68^Ga-P3238 was performed without postlabeling purification steps. The results showed slightly superior results for ^68^Ga-P3246 compared to ^68^Ga-P3238 with regard to cell uptake. Blocking studies with excess folic acid proved FR-specific uptake for both radioconjugates. FR-binding affinities were in the low nanomolar range for both derivatives. Tissue distribution of both ^68^Ga-radioconjugates showed high and FR-specific accumulation in KB tumor xenografts of about 16% ID/g and 15% ID/g at 4 h p.i. In both cases uptake in the kidneys was high and hence the tumor-to-kidney ratio was low (<0.18) at all time-points of investigation [51]. The most remarkable difference among the two derivatives was the uptake in the liver which was significantly higher in the case of ^68^Ga-P3238 (2.49 ± 0.21% ID/g, 4 h p.i.) compared to ^68^Ga-P3246 (1.07 ± 0.18% ID/g, 4 h p.i.). PET imaging studies were performed using a small-animal PET scanner (Focus 120 micro PET scanner, Concorde Microsystems Inc., Knoxville, TN, USA). The imaging results were obtained from *post mortem* scans performed 1 h after injection of 10–12 MBq ^68^Ga-P3246 (0.4 nmol per mouse) with and without pre-injected pemetrexed (Figure 11) [51].

As expected from previous experiments performed by Müller *et al*. [81,82,84,85], application of pemetrexed reduced renal accumulation of radioactivity significantly. Uptake of radioactivity in the gall bladder or intestinal tract was not seen on these images [51]. The authors highlighted the easy preparation of these ^68^Ga-folate conjugates which were accessible at room temperature [51] in contrast to the DOTA-folate conjugates which required radiolabeling at elevated temperatures [50,85]. In addition, the ^68^Ga-NODAGA-conjugates revealed increased tumor-to-blood, tumor-to-muscle and tumor-to-liver ratios compared to the previously established ^68^Ga-DOTA-folates [50,85]. Based on these facts and the favorable *in vivo* characteristics Fani *et al*. proposed ^68^Ga-P3246 as an excellent candidate for clinical application [51].

## 5. [^152^Tb]Terbium-Labeled DOTA-Folate

**Figure 12:**
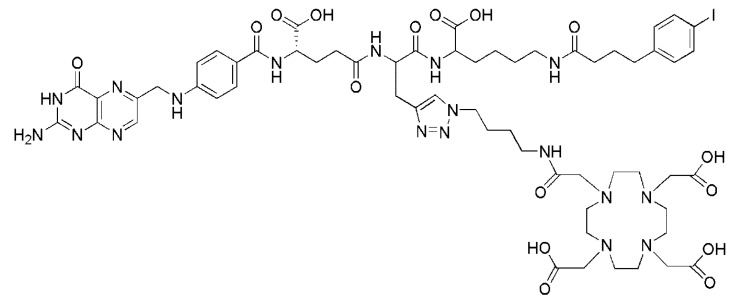
Chemical structure of cm09 composed of three functionalities: folic acid acts as a targeting agent for FR-specific uptake in the tumor tissue; a DOTA-chelator is needed for stable coordination of trivalent radiometals (e.g., ^152^Tb, ^44^Sc); a small molecular weight albumin binding entity is responsible for an enhanced circulation time of the radioconjugate in the blood.

Recently, ^152^Tb was tested *in vivo* by the use of a newly designed DOTA-folate conjugate (cm09, Figure 12) [52]. This folate conjugate was recently tested in its ^177^Lu-labeled version (^177^Lu-cm09) [93]. It comprised an additional functionality which is known to bind to serum albumin with an affinity in the μM-range [94]. Hence, integration of this albumin binding entity into the DOTA-folate molecule’s backbone enhanced the blood circulation time of ^177^Lu-cm09. The result was an increased uptake of ^177^Lu-cm09 in the tumor xenografts and a reduced accumulation in the kidneys compared to other ^177^Lu-labeled DOTA-folate conjugates which lack an albumin binding entity [93].

**Figure 13:**
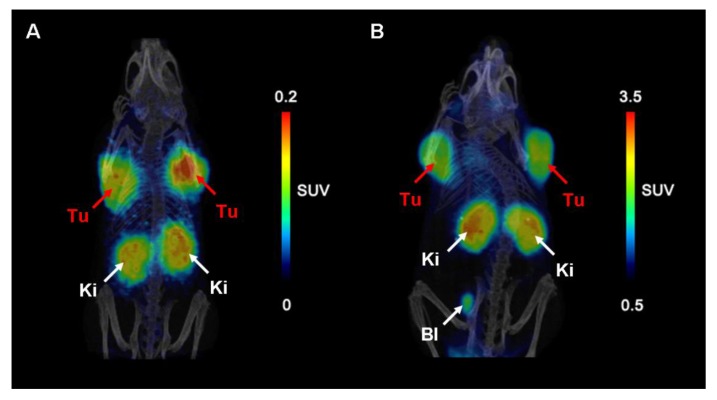
(**A**) *Post mortem* PET/CT image of a KB tumor bearing mouse 24 h after injection of ^152^Tb-cm09 (~10 MBq). (**B**) *In vivo* PET/CT image of a KB tumor bearing mouse 4 h after injection of ^144^Sc-cm09 (~20 MBq).

Radiolabeling of cm09 (15 nmol) with ^152^Tb was accomplished directly in a solution of α-hydroxy-isobutyric acid in which 20 MBq of ^152^Tb were eluted from the cation exchange chromatography column used for isolation/purification of this isotope [52,95]. The reaction mixture was heated for 15 min to obtain ^152^Tb-cm09 in a radiochemical yield of >96%. The *in vitro* evaluation of Tb-cm09 as well as *post mortem* studies in KB tumor bearing nude mice were performed with the longer-lived ^161^Tb isotope (β^−^- and γ-emitter, t_1/2_ = 6.89 d) due to its easier availability compared to ^152^Tb [52]. Imaging studies were performed with a dedicated small-animal PET/CT scanner (eXplore, GE Healthcare). The PET scans of 90 min duration were performed 1.5 h and 3 h after injection of ^152^Tb-cm09 (~10 MBq, 6.8 nmol per mouse) followed by CTs. In addition a 4 h-lasting *post mortem* scan was performed 24 h p.i. of ^152^Tb-cm09 (Figure 13A). In spite of the much higher positron energy of ^152^Tb (E_av_β^+^: 1080 keV, Table 1) compared to ^18^F and ^68^Ga excellent tumor visualization was achieved in mice injected with ^152^Tb-cm09. Besides, accumulation of radioactivity was also found in the kidneys. As expected from previous experiments with ^177^Lu-cm09 [93], the tumor-to-kidney ratio of ^152^Tb-cm09 was almost one. This value was unprecedentedly high compared to the usually low tumor-to-kidney ratios of radiometallated PET folate radiotracers.

## 6. [^44^Sc]Scandium-Labeled DOTA-Folate

*In vivo* PET studies with ^44^Sc were also performed with cm09 (Figure 12) which was also employed for PET imaging with ^152^Tb [52]. The folate conjugate cm09 was mixed with a ^44^Sc solution and incubated at elevated temperature for 15 min. At a specific activity of 7 MBq/nmol the radiochemical yield was >97%. ^44^Sc-cm09 was tested *in vitro* and *in vivo*. Cell uptake and internalization studies with FR-positive KB tumor cells showed FR-specific binding and an internalized fraction which was comparable to the ^177^Lu-labeled version of cm09 [93]. PET/CT scans were performed at 4 h after injection of ^44^Sc-cm09 (~20 MBq per mouse). The PET scan lasted for 30 min followed by a CT. The excellent imaging quality of these PET/CT images allowed visualization of tumor xenografts and kidneys while other organs and tissues did not accumulate the radiotracer (Figure 13B) [53]. An excellent tumor-to-kidney ratio of almost one was achieved similar to the result obtained with ^152^Tb-cm09 (Figure 13B) [52].

## 7. Perspectives for PET Imaging of Inflammation

The focus of this review article was to delineate recent developments in folate-based PET tracer design. Most commonly these folate radiotracers were evaluated in KB tumor bearing mice which emerged as the standard animal model since KB tumor cells express the FR at high levels. However, beside a possible application of folate radioconjugates for imaging purposes of FR-positive cancer, the utility of these radiotracers for imaging of inflammatory diseases deserves further attention. During the course of imaging cancer patients with a radiolabeled folate conjugate, uptake of radioactivity was coincidentally seen in the knee of a patient [22]. Subsequent examination revealed that the patient was suffering from an inflammatory condition in the joints. In pre-clinical studies it was found that folate-based targeting agents were taken up by activated macrophages involved in the inflammatory process of rheumatoid arthritis and that the uptake was mediated via the FR-β [15,30,96]. The principle of using folate radiotracers for targeting activated macrophages has been exemplified not only in rheumatoid arthritis but also in other inflammatory diseases such as osteoarthritis [97], atherosclerosis [98] and infections [99]. For all of these diseases and most probably in many others which involve activated macrophages a clinical folate radiotracer would have a considerable potential as a tool for early diagnosis, staging of the disease and monitoring of the therapy response. Moreover, if FR-β targeted therapies will advance to a clinical application in the future, folate-based imaging may serve for selection of patients who could profit from such novel treatment options [100,101,102,103,104]. Since clinical PET provides higher resolution and sensitivity compared to clinical SPECT introduction of a potent folate-based PET tracer is expected to have a major impact for the management of inflammatory diseases where detection and quantification of even smallest sites of activated macrophages would be accessible.

## 8. Conclusions

Frequent overexpression of the FR on a variety of tumor types makes it attractive for targeted therapies. Hence, folate-based imaging agents may be useful for selection of patients who could profit from such new therapy concepts and for monitoring response to a particular treatment. Once a FR-targeted radionuclide tumor therapy will be established in the future, folate-based imaging agents may also be used for pre-therapeutic dosimetry.

In the course of ^18^F-based folate tracer development in recent years, constant progress has been made with regard to the radiosynthesis and the *in vivo* tissue distribution characteristics of these radiotracers. The most promising candidates for potential clinical application are clearly [^18^F]fluoro-deoxy-glucose-folate and 3'-aza-2'-[^18^F]fluorofolic acid. Moreover, there is a series of different folate conjugates with macrocyclic chelators suitable for radiometallation. Several studies used the short-lived ^68^Ga isotope for radiolabeling of DOTA- and NODAGA-folate conjugates. The resulting PET images of tumor bearing mice were of excellent quality. A recently developed DOTA-folate conjugate comprises an albumin binding entity which is responsible for an enhanced blood circulation time and hence a better tumor-to-kidney ratio. Excellent results in terms of tumor visualization were obtained with this conjugate in combination with ^44^Sc and ^152^Tb whose physical half-lives matched perfectly with the slower kinetics.

We believe that the FR-α is a target of critical value for nuclear imaging through use of folate-based radiotracers as it is not only expressed on several tumor types but reported to correlate with the aggressiveness of these malignancies. Moreover, employment of folate radiopharmaceuticals for imaging of inflammatory diseases by targeting the FR-β on activated macrophages holds promise as a further field of application. The future will show which of the numerous PET folate tracers will be tested in the clinic and which one would finally evolve into the predicted useful tool in nuclear medicine.

## References

[1] Mittra E., Quon A. (2009). Positron emission tomography/computed tomography: The current technology and applications. Radiol. Clin. North Am..

[2] Kramer-Marek G., Capala J. (2012). Can PET imaging facilitate optimization of cancer therapies?. Curr. Pharm. Des..

[3] Gabriel M., Decristoforo C., Kendler D., Dobrozemsky G., Heute D., Uprimny C., Kovacs P., von Guggenberg E., Bale R., Virgolini I.J. (2007). ^68^Ga-DOTA-Tyr^3^-octreotide PET in neuroendocrine tumors: Comparison with somatostatin receptor scintigraphy and CT. J. Nucl. Med..

[4] Haug A.R., Auernhammer C.J., Wängler B., Schmidt G.P., Uebleis C., Goke B., Cumming P., Bartenstein P., Tiling R., Hacker M. (2010). ^68^Ga-DOTATATE PET/CT for the early prediction of response to somatostatin receptor-mediated radionuclide therapy in patients with well-differentiated neuroendocrine tumors. J. Nucl. Med..

[5] Haug A.R., Cindea-Drimus R., Auernhammer C.J., Reincke M., Wängler B., Uebleis C., Schmidt G.P., Goke B., Bartenstein P., Hacker M. (2012). The role of ^68^Ga-DOTATATE PET/CT in suspected neuroendocrine tumors. J. Nucl. Med..

[6] Müller C. (2012). Folate based radiopharmaceuticals for imaging and therapy of cancer and inflammation. Curr. Pharm. Des..

[7] Antony A.C. (1996). Folate receptors. Ann. Rev. Nutr..

[8] Ratnam M., Marquardt H., Duhring J.L., Freisheim J.H. (1989). Homologous membrane folate binding-proteins in human-placenta—Cloning and sequence of a cDNA. Biochemistry.

[9] Shen F., Ross J.F., Wang X., Ratnam M. (1994). Identification of a novel folate receptor, a truncated receptor, and receptor type β in hematopoietic cells: cDNA cloning, expression, immunoreactivity, and tissue specificity. Biochemistry.

[10] Shen F., Wu M., Ross J.F., Miller D., Ratnam M. (1995). Folate receptor type γ is primarily a secretory protein due to lack of an efficient signal for glycosylphosphatidylinositol modification—Protein characterization and cell type specificity. Biochemistry.

[11] Spiegelstein O., Eudy J.D., Finnell R.H. (2000). Identification of two putative novel folate receptor genes in humans and mouse. Gene.

[12] Garin-Chesa P., Campbell I., Saigo P.E., Lewis J.L., Old L.J., Rettig W.J. (1993). Trophoblast and ovarian cancer antigen LK26—Sensitivity and specificity in immunopathology and molecular identification as a folate-binding protein. Am. J. Pathol..

[13] Parker N., Turk M.J., Westrick E., Lewis J.D., Low P.S., Leamon C.P. (2005). Folate receptor expression in carcinomas and normal tissues determined by a quantitative radioligand binding assay. Anal. Biochem..

[14] Low P.S., Kularatne S.A. (2009). Folate-targeted therapeutic and imaging agents for cancer. Curr. Opin. Chem. Biol..

[15] Xia W., Hilgenbrink A.R., Matteson E.L., Lockwood M.B., Cheng J.X., Low P.S. (2009). A functional folate receptor is induced during macrophage activation and can be used to target drugs to activated macrophages. Blood.

[16] Weitman S.D., Lark R.H., Coney L.R., Fort D.W., Frasca V., Zurawski V.R., Kamen B.A. (1992). Distribution of the folate receptor GP38 in normal and malignant cell lines and tissues. Cancer Res..

[17] Weitman S.D., Weinberg A.G., Coney L.R., Zurawski V.R., Jennings D.S., Kamen B.A. (1992). Cellular localization of the folate receptor: Potential role in drug toxicity and folate homeostasis. Cancer Res..

[18] Müller C., Forrer F., Schibli R., Krenning E.P., de Jong M. (2008). SPECT study of folate receptor-positive malignant and normal tissues in mice using a novel ^99m^Tc-radiofolate. J. Nucl. Med..

[19] Holm J., Hansen S.I., Hoiermadsen M., Bostad L. (1992). A high-affinity folate binding-protein in proximal tubule cells of human kidney. Kidney Int..

[20] Sandoval R.M., Kennedy M.D., Low P.S., Molitoris B.A. (2004). Uptake and trafficking of fluorescent conjugates of folic acid in intact kidney determined using intravital two-photon microscopy. Am. J. Physiol. Cell Physiol..

[21] Birn H., Spiegelstein O., Christensen E.I., Finnell R.H. (2005). Renal tubular reabsorption of folate mediated by folate binding protein 1. J. Am. Soc. Nephrol..

[22] Low P.S., Henne W.A., Doorneweerd D.D. (2008). Discovery and development of folic-acid-based receptor targeting for imaging and therapy of cancer and inflammatory diseases. Acc. Chem. Res..

[23] Iwakiri S., Sonobe M., Nagai S., Hirata T., Wada H., Miyahara R. (2008). Expression status of folate receptor alpha is significantly correlated with prognosis in non-small-cell lung cancers. Ann. Surg. Oncol..

[24] Toffoli G., Russo A., Gallo A., Cernigoi C., Miotti S., Sorio R., Tumolo S., Boiocchi M. (1998). Expression of folate binding protein as a prognostic factor for response to platinum-containing chemotherapy and survival in human ovarian cancer. Int. J. Cancer.

[25] Brown Jones M., Neuper C., Clayton A., Mariani A., Konecny G., Thomas M.B., Keeney G., Hartmann L., Podratz K.C. (2008). Rationale for folate receptor alpha targeted therapy in “high risk” endometrial carcinomas. Int. J. Cancer.

[26] Hartmann L.C., Keeney G.L., Lingle W.L., Christianson T.J., Varghese B., Hillman D., Oberg A.L., Low P.S. (2007). Folate receptor overexpression is associated with poor outcome in breast cancer. Int. J. Cancer.

[27] D'Angelica M., Ammori J., Gonen M., Klimstra D.S., Low P.S., Murphy L., Weiser M.R., Paty P.B., Fong Y., Dematteo R.P., Allen P., Jarnagin W.R., Shia J. (2011). Folate receptor-α expression in resectable hepatic colorectal cancer metastases: Patterns and significance. Mod. Pathol..

[28] Kinne R.W., Brauer R., Stuhlmuller B., Palombo-Kinne E., Burmester G.R. (2000). Macrophages in rheumatoid arthritis. Arthritis Res..

[29] Tak P.P., Smeets T.J., Daha M.R., Kluin P.M., Meijers K.A., Brand R., Meinders A.E., Breedveld F.C. (1997). Analysis of the synovial cell infiltrate in early rheumatoid synovial tissue in relation to local disease activity. Arthritis Rheum..

[30] Paulos C.M., Turk M.J., Breur G.J., Low P.S. (2004). Folate receptor-mediated targeting of therapeutic and imaging agents to activated macrophages in rheumatoid arthritis. Adv. Drug Deliv. Rev..

[31] Ke C.Y., Mathias C.J., Green M.A. (2004). Folate-receptor-targeted radionuclide imaging agents. Adv. Drug Deliv. Rev..

[32] Müller C., Schibli R. (2011). Folic acid conjugates for nuclear imaging of folate receptor-positive cancer. J. Nucl. Med..

[33] Siegel B.A., Dehdashti F., Mutch D.G., Podoloff D.A., Wendt R., Sutton G.P., Burt R.W., Ellis P.R., Mathias C.J., Green M.A. (2003). Evaluation of ^111^In-DTPA-folate as a receptor-targeted diagnostic agent for ovarian cancer: Initial clinical results. J. Nucl. Med..

[34] Fisher R.E., Siegel B.A., Edell S.L., Oyesiku N.M., Morgenstern D.E., Messmann R.A., Amato R.J. (2008). Exploratory study of ^99m^Tc-EC20 imaging for identifying patients with folate receptor-positive solid tumors. J. Nucl. Med..

[35] Edelman M.J., Harb W.A., Pal S.E., Boccia R.V., Kraut M.J., Bonomi P., Conley B.A., Rogers J.S., Messmann R.A., Garon E.B. (2012). Multicenter trial of EC145 in advanced, folate-receptor positive adenocarcinoma of the lung. J. Thorac. Oncol..

[36] Jiang L., Zeng X., Wang Z., Chen Q. (2009). Cell line cross-contamination: KB is not an oral squamous cell carcinoma cell line. Eur. J. Oral Sci..

[37] Miller P.W., Long N.J., Vilar R., Gee A.D. (2008). Synthesis of ^11^C, ^18^F, ^15^O, and ^13^N radiolabels for positron emission tomography. Angew. Chem. Int. Ed. Engl..

[38] Antunes P., Ginj M., Zhang H., Waser B., Baum R.P., Reubi J.C., Maecke H. (2007). Are radiogallium-labelled DOTA-conjugated somatostatin analogues superior to those labelled with other radiometals?. Eur. J. Nucl. Med. Mol. Imaging.

[39] Fani M., Andre J.P., Maecke H.R. (2008). ^68^Ga-PET: A powerful generator-based alternative to cyclotron-based PET radiopharmaceuticals. Contrast Media Mol. Imaging.

[40] Zhernosekov K.P., Filosofov D.V., Baum R.P., Aschoff P., Bihl H., Razbash A.A., Jahn M., Jennewein M., Rösch F. (2007). Processing of generator-produced ^68^Ga for medical application. J. Nucl. Med..

[41] Graham M.C., Pentlow K.S., Mawlawi O., Finn R.D., Daghighian F., Larson S.M. (1997). An investigation of the physical characteristics of ^66^Ga as an isotope for PET imaging and quantification. Med. Phys..

[42] Bettio A., Honer M., Müller C., Brühlmeier M., Müller U., Schibli R., Groehn V., Schubiger A.P., Ametamey S.M. (2006). Synthesis and preclinical evaluation of a folic acid derivative labeled with ^18^F for PET imaging of folate receptor-positive tumors. J. Nucl. Med..

[43] Ross T.L., Honer M., Lam P.Y.H., Mindt T.L., Groehn V., Schibli R., Schubiger P.A., Ametamey S.M. (2008). Fluorine-18 click radiosynthesis and preclinical evaluation of a new ^18^F-labeled folic acid derivative. Bioconjug. Chem..

[44] Ross T.L., Honer M., Müller C., Groehn V., Schibli R., Ametamey S.M. (2010). A new ^18^F-labeled folic acid derivative with improved properties for the PET imaging of folate receptor-positive tumors. J. Nucl. Med..

[45] Fischer C.R., Müller C., Reber J., Müller A., Krämer S.D., Ametamey S.M., Schibli R. (2012). [^18^F]fluoro-deoxy-glucose folate: A novel PET radiotracer with improved in vivo properties for folate receptor targeting. Bioconjug. Chem..

[46] Betzel T., Müller C., Groehn V., Müller A., Reber J., Fischer C.R., Krämer S.D., Schibli R., Ametamey S.M. (2013). Radiosynthesis and preclinical evaluation of 3'-aza-2'-[^18^F]fluorofolic acid: A novel PET radiotracer for folate receptor targeting. Bioconjug. Chem..

[47] Mathias C.J., Lewis M.R., Reichert D.E., Laforest R., Sharp T.L., Lewis J.S., Yang Z.F., Waters D.J., Snyder P.W., Low P.S. (2003). Preparation of ^66^Ga- and ^68^Ga-labeled Ga(III)-deferoxamine-folate as potential folate-receptor-targeted PET radiopharmaceuticals. Nucl. Med. Biol..

[48] Mathias C.J., Wang S., Lee R.J., Waters D.J., Low P.S., Green M.A. (1996). Tumor-selective radiopharmaceutical targeting via receptor-mediated endocytosis of gallium-67-deferoxamine-folate. J. Nucl. Med..

[49] Mathias C.J., Wang S., Low P.S., Waters D.J., Green M.A. (1999). Receptor-mediated targeting of ^67^Ga-deferoxamine-folate to folate-receptor-positive human KB tumor xenografts. Nucl. Med. Biol..

[50] Fani M., Wang X., Nicolas G., Medina C., Raynal I., Port M., Maecke H.R. (2011). Development of new folate-based PET radiotracers: Preclinical evaluation of Ga-DOTA-folate conjugates. Eur. J. Nucl. Med. Mol. Imaging.

[51] Fani M., Tamma M.L., Nicolas G.P., Lasri E., Medina C., Raynal I., Port M., Weber W.A., Maecke H.R. (2012). *In vivo* imaging of folate receptor positive tumor xenografts using novel ^68^Ga-NODAGA-folate conjugates. Mol. Pharm..

[52] Müller C., Zhernosekov K., Koster U., Johnston K., Dorrer H., Hohn A., van der Walt N.T., Türler A., Schibli R. (2012). A unique matched quadruplet of terbium radioisotopes for PET and SPECT and for α- and β- radionuclide therapy: An *in vivo* proof-of-concept study with a new receptor-targeted folate derivative. J. Nucl. Med..

[53] Müller C., Bunka M., Reber J., Fischer C., Zhernosekov K., Türler A., Schibli R. Promises of cyclotron produced ^44^Sc as surrogate for ^68^Ga and diagnostic match for ^177^Lu: *In vitro* and *in vivo* study of a ^44^Sc-DOTA-folate conjugate. J. Nucl. Med..

[54] Allen B.J., Goozee G., Sarkar S., Beyer G., Morel C., Byrne A.P. (2001). Production of terbium-152 by heavy ion reactions and proton induced spallation. Appl. Radiat. Isot..

[55] Köster U., SOLDE Collaboration (2001). ISOLDE target and ion source chemistry. Radiochim. Acta.

[56] Majkowska-Pilip A., Bilewicz A. (2011). Macrocyclic complexes of scandium radionuclides as precursors for diagnostic and therapeutic radiopharmaceuticals. J. Inorg. Biochem..

[57] Koumarianou E., Loktionova N.S., Fellner M., Roesch F., Thews O., Pawlak D., Archimandritis S.C., Mikolajczak R. (2012). ^44^Sc-DOTA-BN[2–14]NH_2_ in comparison to ^68^Ga-DOTA-BN[2–14]NH_2_ in pre-clinical investigation. Is ^44^Sc a potential radionuclide for PET?. Appl. Radiat. Isot..

[58] Eigner S., Vera D.R., Fellner M., Loktionova N.S., Piel M., Lebeda O., Rösch F., Ross T.L., Henke K.E. (2013). Imaging of protein synthesis: *In vitro* and *in vivo* evaluation of ^44^Sc-DOTA-puromycin. Mol. Imaging Biol..

[59] Roesch F. (2012). Scandium-44: Benefits of a long-lived PET radionuclide available from the ^44^Ti/^44^Sc generator system. Curr. Radiopharm..

[60] Severin G.W., Engle J.W., Valdovinos H.F., Barnhart T.E., Nickles R.J. (2012). Cyclotron produced ^44g^Sc from natural calcium. Appl. Radiat. Isot..

[61] Dolle F., Hinnen F., Vaufrey F., Tavitian B., Crouzel C. (1997). A general method for labeling oligodeoxynucleotides with ^18^F for *in vivo* PET imaging. J. Labelled Compd. Radiopharm..

[62] Missimer J., Madi Z., Honer M., Keller C., Schubiger A., Ametamey S.M. (2004). Performance evaluation of the 16-module quad-HIDAC small animal PET camera. Phys. Med. Biol..

[63] Al Jammaz I., Al-Otaibi B., Okarvi S., Amartey J. (2006). Novel synthesis of [^18^F]fluorobenzene and pyridinecarbohydrazide-folates as potential PET radiopharmaceuticals. J. Labelled Compd. Radiopharm..

[64] Amartey J.K., Al-Jammaz I., Al-Otaibi B., Esguerra C. (2002). Novel synthesis of 2-[^18^F]fluoroisonicotinic acid hydrazide and initial biological evaluation. Nucl. Med. Biol..

[65] Wang S., Luo J., Lantrip D.A., Waters D.J., Mathias C.J., Green M.A., Fuchs P.L., Low P.S. (1997). Design and synthesis of [^111^In]DTPA-folate for use as a tumor-targeted radiopharmaceutical. Bioconjug. Chem..

[66] Guo W.J., Hinkle G.H., Lee R.J. (1999). ^99m^Tc-HYNIC-folate: A novel receptor-based targeted radiopharmaceutical for tumor imaging. J. Nucl. Med..

[67] Al Jammaz I., Al-Otaibi B., Amer S., Okarvi S.M. (2011). Rapid synthesis and *in vitro* and *in vivo* evaluation of folic acid derivatives labeled with fluorine-18 for PET imaging of folate receptor-positive tumors. Nucl. Med. Biol..

[68] Westerhof G.R., Schornagel J.H., Kathmann I., Jackman A.L., Rosowsky A., Forsch R.A., Hynes J.B., Boyle F.T., Peters G.J., Pinedo H.M. (1995). Carrier- and receptor-mediated transport of folate antagonists targeting folate-dependent enzymes: Correlates of molecular-structure and biological activity. Mol. Pharmacol..

[69] Tornoe C.W., Christensen C., Meldal M. (2002). Peptidotriazoles on solid phase: [1,2,3]-Triazoles by regiospecific copper(I)-catalyzed 1,3-dipolar cycloadditions of terminal alkynes to azides. J. Org. Chem..

[70] Rostovtsev V.V., Green L.G., Fokin V.V., Sharpless K.B. (2002). A stepwise huisgen cycloaddition process: Copper(I)-catalyzed regioselective “ligation” of azides and terminal alkynes. Angew. Chem. Int. Ed. Engl..

[71] Marik J., Sutcliffe J.L. (2006). Click for PET: Rapid preparation of [^18^F]fluoropeptides using CuI catalyzed 1,3-dipolar cycloaddition. Tetrahedron Lett..

[72] Mindt T.L., Müller C., Melis M., de Jong M., Schibli R. (2008). "Click-to-chelate”: *In vitro* and *in vivo* comparison of a ^99m^Tc(CO)_3_-labeled *N*(tau)-histidine folate derivative with its isostructural, clicked 1,2,3-triazole analogue. Bioconjug. Chem..

[73] Mindt T.L., Müller C., Stuker F., Salazar J.F., Hohn A., Müggler T., Rudin M., Schibli R. (2009). A “click chemistry” approach to the efficient synthesis of multiple imaging probes derived from a single precursor. Bioconjug. Chem..

[74] Al Jammaz I., Al-Otaibi B., Amer S., Al-Hokbany N., Okarvi S. (2012). Novel synthesis and preclinical evaluation of folic acid derivatives labeled with [^18^F]FDG for PET imaging of folate receptor-positive tumors. Nucl. Med. Biol..

[75] Ilgan S., Yang D.J., Higuchi T., Zareneyrizi F., Kim E.E., Podoloff D.A. (1998). Imaging tumor folate receptors using ^111^In-DTPA-methotrexate. Cancer Biother. Radiopharm..

[76] Maschauer S., Prante O. (2009). A series of 2-*O*-trifluoromethylsulfonyl-D-mannopyranosides as precursors for concomitant ^18^F-labeling and glycosylation by click chemistry. Carbohydr. Res..

[77] Maschauer S., Einsiedel J., Haubner R., Hocke C., Ocker M., Hubner H., Kuwert T., Gmeiner P., Prante O. (2010). Labeling and glycosylation of peptides using click chemistry: A general approach to ^18^F-glycopeptides as effective imaging probes for positron emission tomography. Angew. Chem. Int. Ed. Engl..

[78] Groehn V., Moser R., Ross T.L., Betzel T., Müller C., Schibli R., Ametamey S. (2011). Synthesis of precursors for ^18^F-labeling of folic acid for PET application. Synthesis.

[79] Curtin N.J., Hughes A.N. (2001). Pemetrexed disodium, a novel antifolate with multiple targets. Lancet Oncol..

[80] Paz-Ares L., Bezares S., Tabernero J.M., Castellanos D., Cortes-Funes H. (2003). Review of a promising new agent—Pemetrexed disodium. Cancer.

[81] Müller C., Brühlmeier M., Schubiger A.P., Schibli R. (2006). Effects of antifolate drugs on the cellular uptake of radiofolates *in vitro* and *in vivo*. J. Nucl. Med..

[82] Müller C., Schibli R., Krenning E.P., de Jong M. (2008). Pemetrexed improves tumor selectivity of ^111^In-DTPA-folate in mice with folate receptor-positive ovarian cancer. J. Nucl. Med..

[83] Müller C., Reddy J.A., Leamon C.P., Schibli R. (2010). Effects of the antifolates pemetrexed and CB3717 on the tissue distribution of ^99m^Tc-EC20 in xenografted and syngeneic tumor-bearing mice. Mol. Pharm..

[84] Müller C., Mindt T.L., de Jong M., Schibli R. (2009). Evaluation of a novel radiofolate in tumour-bearing mice: Promising prospects for folate-based radionuclide therapy. Eur. J. Nucl. Med. Mol. Imaging.

[85] Müller C., Vlahov I.R., Santhapuram H.K., Leamon C.P., Schibli R. (2011). Tumor targeting using ^67^Ga-DOTA-Bz-folate—Investigations of methods to improve the tissue distribution of radiofolates. Nucl. Med. Biol..

[86] Kühle B., Müller C., Ross T.L. (2013). A novel ^68^Ga-labeled pteroic acid-based PET tracer for tumor imaging via the folate receptor. Recent Results Cancer Res..

[87] Ke C.Y., Mathias C.J., Green M.A. (2005). Targeting the tumor-associated folate receptor with an ^111^In-DTPA conjugate of pteroic acid. J. Am. Chem. Soc..

[88] Müller C., Hohn A., Schubiger P.A., Schibli R. (2006). Preclinical evaluation of novel organometallic ^99m^Tc-folate and ^99m^Tc-pteroate radiotracers for folate receptor-positive tumour targeting. Eur. J. Nucl. Med. Mol Imaging.

[89] Guo H., Xie F., Zhu M., Li Y., Yang Z., Wang X., Lu J. (2011). The synthesis of pteroyl-lys conjugates and its application as technetium-99m labeled radiotracer for folate receptor-positive tumor targeting. Bioorg. Med. Chem. Lett..

[90] Leamon C.P., DePrince R.B., Hendren R.W. (1999). Folate-mediated drug delivery: Effect of alternative conjugation chemistry. J. Drug Targeting.

[91] Leamon C.P., You F., Santhapuram H.K., Fan M., Vlahov I.R. (2009). Properties influencing the relative binding affinity of pteroate derivatives and drug conjugates thereof to the folate receptor. Pharm. Res..

[92] Theti D.S., Bavetsias V., Skelton L.A., Titley J., Gibbs D., Jansen G., Jackman A.L. (2003). Selective delivery of CB300638, a cyclopenta[*g*]quinazoline-based thymidylate synthase inhibitor into human tumor cell lines overexpressing the alpha-isoform of the folate receptor. Cancer Res..

[93] Müller C., Struthers H., Winiger C., Zhernosekov K., Schibli R. (2013). DOTA conjugate with an albumin-binding entity enables the first folic acid-targeted ^177^Lu-radionuclide tumor therapy in mice. J. Nucl. Med..

[94] Dumelin C.E., Trüssel S., Buller F., Trachsel E., Bootz F., Zhang Y., Mannocci L., Beck S.C., Drumea-Mirancea M., Seeliger M.W. (2008). A portable albumin binder from a DNA-encoded chemical library. Angew. Chem. Int. Ed. Engl..

[95] Lehenberger S., Barkhausen C., Cohrs S., Fischer E., Grünberg J., Hohn A., Köster U., Schibli R., Türler A., Zhernosekov K. (2011). The low-energy β− and electron emitter ^161^Tb as an alternative to ^177^Lu for targeted radionuclide therapy. Nucl. Med. Biol..

[96] Turk M.J., Breur G.J., Widmer W.R., Paulos C.M., Xu L.C., Grote L.A., Low P.S. (2002). Folate-targeted imaging of activated macrophages in rats with adjuvant-induced arthritis. Arthritis Rheum..

[97] Piscaer T.M., Müller C., Mindt T.L., Lubberts E., Verhaar J.A., Krenning E.P., Schibli R., de Jong M., Weinans H. (2011). Imaging of activated macrophages in experimental osteoarthritis using folate-targeted animal single-photon-emission computed tomography/computed tomography. Arthritis Rheum..

[98] Ayala-Lopez W., Xia W., Varghese B., Low P.S. (2010). Imaging of atherosclerosis in apoliprotein E knockout mice: Targeting of a folate-conjugated radiopharmaceutical to activated macrophages. J. Nucl. Med..

[99] Henne W.A., Rothenbuhler R., Ayala-Lopez W., Xia W., Varghese B., Low P.S. (2012). Imaging sites of infection using a ^99m^Tc-labeled folate conjugate targeted to folate receptor positive macrophages. Mol. Pharm..

[100] Yi Y.S., Ayala-Lopez W., Kularatne S.A., Low P.S. (2009). Folate-targeted hapten immunotherapy of adjuvant-induced arthritis: Comparison of hapten potencies. Mol. Pharm..

[101] Lu Y., Stinnette T.W., Westrick E., Klein P.J., Gehrke M.A., Cross V.A., Vlahov I.R., Low P.S., Leamon C.P. (2011). Treatment of experimental adjuvant arthritis with a novel folate receptor-targeted folic acid-aminopterin conjugate. Arthritis Res. Ther..

[102] Henne W. A., Kularatne S. A., Ayala-Lopez W., Doorneweerd D.D., Stinnette T.W., Lu Y., Low P.S. (2012). Synthesis and activity of folate conjugated didemnin B for potential treatment of inflammatory diseases. Bioorg. Med. Chem. Lett..

[103] Nagai T., Kyo A., Hasui K., Takao S., Matsuyama T. (2012). Efficacy of an immunotoxin to folate receptor beta in the intra-articular treatment of antigen-induced arthritis. Arthritis Res. Ther..

[104] Feng Y., Shen J., Streaker E.D., Lockwood M., Zhu Z., Low P.S., Dimitrov D.S. (2011). A folate receptor beta-specific human monoclonal antibody recognizes activated macrophage of rheumatoid patients and mediates antibody-dependent cell-mediated cytotoxicity. Arthritis Res. Ther..

